# Urinary Peptidomic Biomarkers in Kidney Diseases

**DOI:** 10.3390/ijms21010096

**Published:** 2019-12-21

**Authors:** Vittorio Sirolli, Luisa Pieroni, Lorenzo Di Liberato, Andrea Urbani, Mario Bonomini

**Affiliations:** 1 Nephrology and Dialysis Unit, Department of Medicine, G. d’Annunzio University, Chieti-Pescara, SS.Annunziata Hospital, Via dei Vestini, 66013 Chieti, Italy; vittorio.sirolli@unich.it (V.S.); lorenzo.diliberato@asl2abruzzo.it (L.D.L.); 2 Proteomics and Metabonomics Unit, IRCCS Fondazione Santa Lucia, 00179 Rome, Italy; l.pieroni@hsantalucia.it; 3 Institute of Biochemistry and Clinical Biochemistry, Università Cattolica del Sacro Cuore, 00168 Rome, Italy; 4 Department of Laboratory Diagnostic and Infectious Diseases, Fondazione Policlinico Universitario Agostino Gemelli IRCCS, 00168 Rome, Italy

**Keywords:** kidney, urine, peptidomics, biomarker, CKD, clinical proteomics, mass spectrometry

## Abstract

In order to effectively develop personalized medicine for kidney diseases we urgently need to develop highly accurate biomarkers for use in the clinic, since current biomarkers of kidney damage (changes in serum creatinine and/or urine albumin excretion) apply to a later stage of disease, lack accuracy, and are not connected with molecular pathophysiology. Analysis of urine peptide content (urinary peptidomics) has emerged as one of the most attractive areas in disease biomarker discovery. Urinary peptidome analysis allows the detection of short and long-term physiological or pathological changes occurring within the kidney. Urinary peptidomics has been applied extensively for several years now in renal patients, and may greatly improve kidney disease management by supporting earlier and more accurate detection, prognostic assessment, and prediction of response to treatment. It also promises better understanding of kidney disease pathophysiology, and has been proposed as a “liquid biopsy” to discriminate various types of renal disorders. Furthermore, proteins being the major drug targets, peptidome analysis may allow one to evaluate the effects of therapies at the protein signaling pathway level. We here review the most recent findings on urinary peptidomics in the setting of the most common kidney diseases.

## 1. Introduction

Kidney diseases can be caused by a variety of insults such as hypertension, genetic or metabolic disorders, infections, toxins, ischemia, immunological disorders or allograft rejection. These renal insults may promote the development of chronic kidney disease (CKD), a major health problem affecting ~ 10% of the general population and causing a huge economic and personal burden [1].

In clinical practice, kidney damage is generally detected by changes in serum creatinine and a creatinine-based estimate of the glomerular filtration rate (eGFR), and/or urinary albumin/protein excretion. However, both methods have major limitations, including nonspecificity, substantial variability, and lack of accuracy [2]. A significant increase in serum creatinine concentration indicates that more than 50% of the glomerular function has been lost, a stage of substantial irreversible damage. Albuminuria may represent an early marker of renal injury proceeding a decline in renal function. However, it is not able to distinguish different types of proteinuric kidney disease and has a limited ability in the prediction of disease progression and determination of therapeutic efficacy. In addition, neither marker is connected with molecular pathophysiology, but rather represents a consequence of substantial damage to the kidney. Thus, there is the unmet need for new biomarkers enabling a more accurate and timely detection of kidney dysfunction, which in turn may lead to an improvement in both short- and long-term outcomes. Early intervention is indeed the most effective approach [3].

Urinary proteomics has progressively emerged as one of the most attractive topics in disease biomarker discovery [4]. The proteome comprises the entire set of proteins expressed in a biological sample. Urine is a valuable biofluid for the discovery of disease biomarkers, since collecting it is simple, noninvasive, readily available, and in relatively abundant volume. Approximately 70% of the urinary proteome is from the urogenital tract, with the remaining 30% expected to be of systemic origin. The urinary proteome is close to the clinical phenotype and, representing both acute and long-term events, probably delivers the most stable readout on the kidney [5]. Moreover, proteins are the major drug targets. Thus, urinary proteomics has been applied extensively these last several years, with a view to identifying markers of kidney disease progression, diagnosis, or responsiveness to therapy [2,4,6,7,8].

Both proteins and peptides (short aminoacid chains) are contained in urine but peptides are mainly the results of complex molecular post-translational modifications of larger polypeptide molecules. In fact, their specific structures are not only representative of a single gene product, but effectively represent the action of multiple biochemical steps. Peptidomics focuses on the discovery and quantitation of such endogenous peptides, given their wide range of encoded biological functions and their associated diagnostic and therapeutic potential [9]. Endogenous urinary peptides are favored over urinary full-length proteins as noninvasive biomarkers of kidney disease, for several reasons [5]. These include: the fact that, being filtered by the kidney and excreted under physiological conditions, urinary peptide can show changes in content before failure of the glomerular filtration barrier (in contrast to proteins); the higher stability of the urinary peptidome, which is less amenable to damage in the bladder than the urinary proteome; and the possibility of direct mass spectrometry analysis without the tryptic digestion that is required for urinary protein before analysis and that introduces additional variability [5]. In addition, it has become clear that the combination of peptides in multimarker signatures yields a much more detailed assessment of the disease status than single biomarkers [10].

In this article, after a brief section on available technologies, we review the most recent findings on urinary peptidomics in the setting of the most common kidney diseases, covering the publication years 2005–2019.

## 2. Techniques for Urinary Peptidome Analysis

In contrast to proteomics, that pursues the identification of proteins through the analysis of proteolityc peptides obtained upon enzymatic digestion (bottom up) or entire proteins (top down), peptidomics analysis implies the characterization of the complete collection of naturally occurring peptides present in the sample without any preventive protein digestion. Pros and cons can be defined for both these approaches [11].

For peptides analysis, technologies that can assure limited pre-analytical manipulation, low dynamic range of detection, high mass accuracy, high reproducibility and rapid analysis times are required. The complexity of the chemical properties of the peptidome has been only partially addressed mainly due to the large chemical space in which this class of molecules falls. Multiple analytical separation techniques have been employed including nano- and microscale ultra performance liquid chromatography (UPLC) and capillary electrophoresis (CE) in combination with mass spectrometry (MS).

Mass spectrometry is an analytical technique that allows the characterization of mass, structure and concentration of unknown molecules, provided that molecules are in their ionized form. Ionized molecules in gas phase pass through a mass analyzer and their mass over charge ratio is measured as a function of their relative abundance. Peptides ionization step prior to MS scan can be performed by means of Electrospray Ionization (ESI) or Matrix Assisted Laser Desorption Ionization (MALDI).

CE-MS has been widely used for urine peptidome analysis. The separation of sample in CE is based on the assumption that the electrophoretic mobility of the molecule is directly proportional to its charge and inversely proportional to its size. The application of a high voltage to a capillary filled with an electrolyte solution, generates both an electrosmotic flow and an electrophoretic migration, allowing to separate the analytes with a high resolution. Before CE peptide separation, sample needs to pass through some pre-analytical manipulation, to reduce its complexity and to eliminate contaminant compounds. Several methods have been developed to prepare native peptides from urine samples [12].

CE, which is commonly coupled with an ESI ion source, in comparison with LC, shows some advantages: it allows the separation of small multicharged peptides, which are poorly retained by the stationary phases commonly used in the reverse phase column used in LC, or can separate large molecules which are not efficiently eluted from the LC column [13]. Moreover, being more sensitive to background interference, LC-MS requires more intensive purification step of the sample, which is commonly achieved applying specific solid phase extraction (SPE) methods to clean native peptides from the urine matrix [14]. A CE–MS weakness is the reduced possibility to identify peptides, which is achieved upon LC-MS analysis. Nonetheless, in general, LC and CE separation give similar result performances in terms of number and mass range of detected peptides when coupled to ESI-MS.

Direct analysis by MALDI TOF MS has also been used for urinary peptide profiling. The advantage is that it does not require preventive purification step and that all the peptides are simultaneously evidenced in the detection window. It represents an attractive high throughput platform for urine biomarker discovery, though particular care is required in sample manipulation to reduce pre-analytical and analytical variability [15]. In MALDI TOF MS it is difficult to immediately identify detected pick, but the identification and characterization of the urinary peptides can be obtained by the TOF-TOF apparatus and the application of recent bioinformatics procedures. MALDI-MS gives the possibility to detect large panels of biomarkers, including low molecular weight and polar peptides.

Lastly, though MALDI reproducibility may be affected by different sources of measurement error, the application of biostatistical tools during the data analysis can improve the reliability of the instrumental measurement [16].

The analytical technologies so far applied are still in a framework of research application only. Validation of these mass spectrometer platforms, as observed in other applications [17], is still missing and it would be highly required in order not to turn back to immunochemistry-based tests. These assays may well suffer from the lack of specificity of the employed antibody for a unique peptide molecular species, thus providing an indirect response on a plethora of molecular species sharing the same epitope structure.

## 3. Clinical Applications of Urinary Peptidomics in Kidney Disease

### 3.1. Chronic Kidney Disease

CKD is defined as structural renal damage, or as a glomerular filtration rate (GFR) < 60 mL/min/1.73 m^2^ for at least three months [1]. CKD patients represent the largest population in kidney disease, and most urinary peptidomic studies have been performed in such patients, seeking to improve noninvasive CKD detection and progression.

One large study involved 379 healthy subjects and 230 patients suffering from various kidney diseases identified by CE-MS 273 peptides that significantly distinguished between CKD and healthy controls [13]. These 273 peptides were combined into a classifier termed CKD273 using a support-vector machine. The CKD273 classifier showed a sensitivity of 85% and a specificity of 100% with an area under the curve (AUC) of 0.96 for the correct diagnosis of CKD. Similar accuracy in the diagnosis of CKD was confirmed in later studies [2].

Beside detection of CKD, application of the CKD273 classifier was also found to enable prediction of progression of kidney disease in patients with CKD of various etiologies [18,19,20]. The validity and value of CKD273 classifier for predicting CKD progression was confirmed in a systematic review applying the Oxford Evidence-Based Medicine and Southampton Oxford Retrieval Team guidelines [21]. In 522 patients with a follow-up of 54 ± 28 months from a cohort of 1767 CKD patients (52.9% diabetics), CKD273 classifier displayed significantly better predictive value (AUC 0.831) for progression of kidney disease as evidenced by decreased eGFR, than did the combination of eGFR + urinary albumin (AUC 0.758) [19]. Pontillo et al. [22] collected samples from 2672 patients (75% diabetics) at different CKD stages, aiming to predict eGFR loss at different baseline GFR strata. In early stages of disease (eGFR > 70 mL/min/1.73 m^2^), the CKD273 analyzer outperformed albuminuria in predicting eGFR loss, whereas albuminuria performed better in CKD subjects with late-stage disease (eGFR < 50 mL/min/1.73 m^2^). In a longitudinal study including 2087 patients with baseline eGFR > 60 mL/min/1.73 m^2^, over a median 4.6 years, CKD273 improved prediction of decline in glomerular filtration and progression to CKD stage 3 (eGFR < 60 mL/min/1.73 m^2^), while accounting for baseline eGFR, albuminuria and covariables [23]. Note that in 2016, the European Medicines Agency [24] proposed that progression to CKD stage 3 represents an efficacy endpoint for showing significant benefit in primary prevention of renal outcome. Taken together, these results suggest that the peptide pattern may be used in clinical practice to identify patients at elevated risk of progression and needing optimal renoprotective treatment [3]. Furthermore, in 49 patients with different stages of CKD, over 3.6 years of follow-up subjects reaching an endpoint (starting dialysis, *n* = 9; death, *n* = 6) displayed a much higher CKD273 score than subjects alive and not dialyzed [20].

In the CKD273 classifier, the sequenced markers for the diagnosis of CKD [13] were diverse collagen fragments (most of them downregulated), blood and kidney-specific proteins, as well as, fragments of various secreted proteins (upregulated. Urinary peptidomics may offer an opportunity for better understanding of CKD pathophysiology. An early decrease in urinary abundance of collagen I, III, and IV fragments is a recurrent finding in peptidome studies in kidney disease. This observation indicates attenuation of collagen degradation in the kidney, which results in an increase of extracellular matrix causing renal interstitial fibrosis, a predictor of the decline in renal function [25]. Collagen fragments represent the most abundant peptides in the urine and are thought to result from proteolytic activity [26]. Collagen breakdown in CKD patients might be inhibited by elevated levels of tissue inhibitor of matrix metalloproteinase type 1, and by reduced levels of alpha-1 antithrypsin [27]. Thus, alterations to extracellular matrix, reflected via the urinary peptidome, may signal an early stage in the pathology of CKD, and preventing the pathological accumulation of extracellular matrix may prove a valuable therapeutic approach in CKD [28]. Furthermore, urinary peptides may be used to diagnose and monitor renal fibrosis. Magalhaes et al. [29] showed a significant and positive correlation between the urinary classifier CKD273 and the degree of fibrosis in 42 kidney biopsies, which could not be detected by the serum and urine biochemical parameters routinely used to estimate the severity of CKD. Seven fibrosis-associated collagen fragments displayed a negative association with the degree of fibrosis, which suggests they have a causal relation to the accumulation of extracellular matrix observed in renal tissue [29]. Interestingly, some CKD-associated abnormalities such as reduction of collagen fragments were found to correlate with aging in adults [30], suggesting a reduced turnover of extracellular matrix promoting fibrosis as a major mechanism of kidney aging.

Several studies have demonstrated the validity of urinary peptidome analysis in the setting of diabetes mellitus, the leading cause of CKD worldwide. In a 5-year follow-up study in 35 normoalbuminuric diabetic patients (type 1, *n* = 16; type 2, *n* = 19), application of the CKD273 classifier led to earlier detection (1.5 years) of progression to macroalbuminuria (AUC 0.93) than did routinely used urinary albumin (AUC 0.67); the prominent biomarkers before onset of macroalbuminuria were decreased collagen fragments [31]. A *post hoc* analysis of 737 samples from the DIRECT (Diabetic Retinopathy Candesartan Trial) 2 study cohort showed the better predictive role of CKD273 over albuminuria in the development of microalbuminuria from a baseline normoalbuminuric state [32]. Demonstration of prediction of progression by CKD273 was also very recently shown in a large study involving more than 1000 diabetic patients with no evidence of kidney disease [33]. Moreover, in 155 patients with type 2 diabetes and microalbuminuria, the CKD273 classifier score was significantly associated over a 6-year follow-up period with all-cause mortality (log risk [Mantel-Cox] *p* = 0.004), and retained significance after adjustment for sex, age, blood pressure, coronary artery calcium score, and N-terminal pro-brain natriuretic peptide [34].

Apart from diagnostic and prognostic value, analysis of urinary polypeptide patterns in diabetics has also shown its potential in assessing therapeutic intervention. Eighteen type 2 diabetic patients with diabetic retinopathy and macroalbuminuria (≥300 mg/die) were studied in a randomized double-blinded, cross-over trial including four treatment periods each lasting two months [35]. Each patient received, in random order, placebo and the angiotensin II receptor antagonist candesartan (8, 16, and 32 mg) once daily. At the end of each treatment period urinary polypeptide patterns were evaluated by CE-MS. Treatment effects were examined by changes in the diabetic renal damage pattern, composed of 113 polypeptides (11 identified, including fragments of albumin, collagen, and Tamm-Horsfall protein) differing in frequency of occurrence and abundance between normoalbuminuric and macroalbuminuric patients. Upon treatment with candesartan, 15 of the 113 diabetic renal damage markers were significantly changed. Changes were characterized by a combined reduction of disease-specific and an increase of normal-specific signals, toward levels found in normoalbuminuric patients. The favorable effect of candesartan was not dose-dependent but it was noted that individual changes correlated with changes in albuminuria at each dose level: the greater the decline in diabetic renal damage pattern, the greater the decline in albuminuria [35]. In patients treated with another angiotensin receptor blocker (irbesartan), a change in CKD273 scores toward values of healthy individuals was detected after two years of treatment [36]. Several peptides showed significant variation, including increased collagen fragments. More recently, the predictivity of CKD273 on albuminuria response to treatment with the mineralcorticoid receptor inhibitor, spironolactone was evaluated in a *post hoc* analysis of a double-blind randomized clinical trial [37]. Patients with type 2 diabetes and resistant hypertension were allocated to receive for 16 weeks either placebo (*n* = 54) or spironolactone (12.5–50 mg/day; *n* = 57) as an adjunct to inhibition of the renin-angiotensin system. The percentage change in the urine albumin to creatinine ratio (UACR) represented the primary end-point of the study. The authors found that higher values of CKD273 at baseline were associated with a larger decrease in UACR in patients treated with spironolactone (*p* = 0.049), but not in the placebo group (*p* = 0.25). After stratification into tertiles of baseline CKD273 score, a significant reduction in UACR during treatment (63%; *p* = 0.013) was found in the highest tertile only; the reduction there was significantly higher (*p* = 0.013) than in the other two tertiles combined. These results suggest that the urinary proteomics classifier may represent a valuable tool for tailoring therapy, distinguishing between those diabetic patients at greatest risk of progression and those with the best albuminuria-lowering response to treatment with spironolactone [37].

The combination of risk assessment and treatment benefit with spironolactone was tested in the PRIORITY trial. The Proteomic prediction and Renin angiotensin aldosterone system Inhibition prevention of early diabetic nephRopathy in TYpe 2 diabetic patients with normoalbuminuria (PRIORITY) trial (NCT02040441) was a randomized, double-blind, placebo-controlled multicenter clinical trial and observational study in diabetics with eGFR > 45 mL/min/1.73 m^2^ and normoalbuminuria, funded by the European Union [38]. Participants were stratified as high- or low-risk based on a CKD273 score > 0.154 or ≤ 0.154, respectively. Of the 1775 adult patients enrolled, 216 were in the high-risk group for developing microabuminuria. High-risk patients were then randomized to either spironolactone 25 mg once daily (*n* = 102) or placebo (*n* = 107) on top of standard care for 2.5 years. Standard care was continued in the low-risk group. The aims of the PRIORITY study were (I) to test CKD273 prospectively as predictive of development of microalbuminuria (primary end-point), and (II) to evaluate in subjects with a high-risk score whether intervention with spironolactone can reduce the risk of microalbuminuria. Traditional diabetic disease risk factors differed slightly between participants at high risk and those at low-risk of disease progression based on the CKD273 score, suggesting that the classifier may provide additional prognostic information over and above the clinical data [39]. Results of the trial have been presented very recently [40]. During 4.5 years of follow-up, high-risk proteomic pattern patients had a 2.5 fold higher risk of developing microalbuminuria, and a significantly higher risk of developing CKD type 3 or worse (*p* < 0.0001). However, there was no difference in the occurrence of new kidney disease between patients treated with spironolactone or placebo (HR 0.81, *p* = 0.41), which might be related to the study design or to spironolactone only having an effect at more advanced stages of kidney damage [40].

Stratification of CKD patients by CKD273 classifier was also recently investigated in urine samples derived from the completed MARLINA-T2D clinical trial [41]. The Efficacy, Safety & Modification of Albuminuria in type 2 Diabetes Subjects with Renal Disease with LINAgliptin (MARLINA-T2D; NCT 01792518) was a randomized clinical trial evaluating (and showing) the glycemic efficacy of the dipeptidyl peptidase-4 inhibitor, linagliptin (5 mg daily for 24 weeks), on top of recommended standard care for diabetic kidney disease [42]. In their exploratory study, Siwy et al. [41] found that, while no differences in eGFR were observed in the overall cohort of patients treated with linagliptin as compared to placebo, use of CKD273 classification allowed the stratification of patients into high-risk (baseline CKD273 score > 0.343) and low-risk of progression. In high-risk patients, treatment with linagliptin prevented a significant loss of renal function, as observed in placebo-treated high-risk subjects. No differences occurred between linagliptin and placebo in the low-risk groups. In addition, the urinary peptide pattern disclosed dynamic changes upon treatment with linagliptin (993 potentially affected peptides), which may indicate that the drug inhibits selective proteases within the kidney, something that might be used to assess compliance and monitor therapy. A previous *post-hoc* analysis in 40 uncomplicated type 1 diabetics treated for 8 weeks with the sodium glucose cotransporter 2 inhibitor, empagliflozin, showed that treatment significantly changed the amount of 107 peptides in the urine, the direction being toward “CKD absent” [43]. These observations [41,43] suggest a potential nephroprotective effect impacting the urinary peptidome by modern antiglycemic treatments, which merits further investigation.

More recently, CKD subclassifiers specific for CKD stages were generated, allowing early identification of patients at high risk of CKD progression [44]. Urinary peptidomics CKD273 subclassifiers outperformed CKD classifier and urinary albumin for predicting rapid loss of eGFR in individuals with baseline eGFR > 60 mL/min/1.73 m^2^, and even predicted a rapid CKD progression in individuals with eGFR > 60 mL/min/1.73 m^2^ and albuminuria < 30 mg/day. The association between CKD273 subclassifiers and rapid CKD progression remained significant after adjustment for sex, age, diabetes, albuminuria, baseline eGFR, and systolic blood pressure. In rapid progressors with eGFR > 60 mL/min/1.73 m^2^, down-regulated collagen fragments were the predominant differentially expressed peptides, whereas in more advanced CKD, peptides deriving from plasma proteins were abundant and associated with faster progression. When peptide overlap with CKD273 was examined, common peptides varied according to the eGFR stratum. The highest number of common peptides was found for stratum 1 (eGFR ≥ 80 mL/min/1.73 m^2^), whereas in stratum 6 (eGFR 30–39 mL/min/1.73 m^2^) there was no peptide overlapping [44].

A different approach to evaluating CKD progression was used in a recent study which focused on investigating molecular alterations in CKD patients presenting an improved GFR [45]. The fact is that, though usually characterized by a progressive decline in GFR over time, CKD may also in some cases show a tendency toward stabilization or even improvement of renal function (increased GFR slope) as a possible clinical outcome, whose underlying reasons or predictors remain unclear. Data from 553 analyzed urine samples from the Human Urinary Proteome database [46] were used to stratify CKD patients with a baseline eGFR ≥ 15 mL/min/1.73 m^2^ and at least 3 years of follow-up into non-progressors or stable (eGFR slope/year between −1.5% and +1.5%; *n* = 376) as opposed to those with an improved eGFR (slope/year > 5%; *n* = 177) [45]. Out of 384 peptides that significantly differed between the two study populations, 141 sequenced peptide fragments were used to generate a support vector machine-based classifier which resulted in an AUC value of 0.85 along with specificity and sensitivity of 81%. Most peptides (78%) with significantly altered levels in patients with an improvement in renal function belonged to different forms of collagen (66 downregulated, 44 upregulated). However, such collagen fragments differed from those of the panel associated with the process of progression [19]. Only 29 peptides in fact were found in common with the CKD273 classifier, which as expected poorly identified patients with improved eGFR [45]. These results suggest that CKD patients with improving renal function undergo different biological and/or molecular processes compared to CKD progressors, which may ultimately lead to the identification of new molecular targets for CKD remission [45]. In addition, this prognostic classifier might help indicate patients predisposed to an improvement in renal function and thus avoid unnecessary medical intervention in low-risk patients.

In summary, the available evidence underscores the utility of urinary peptidomics in the detection and stratification of patients suffering from CKD. Also, peptides identified by the peptidome analysis may point to abnormal biological pathways and hence lead to new therapeutic targets.

### 3.2. Acute Kidney Injury

Acute kidney injury (AKI) is defined as a rapid decrease in glomerular function and/or urine output. AKI is the most frequent acute renal condition, is associated with significant morbidity and mortality, and increases the risk of CKD [47,48].

Current diagnostic tools do not allow early detection or prediction of the course of AKI, thereby hampering any improvement in patient outcome [49]. The search for biomarkers predicting progression to severe damage has come up with substances that exhibit the presence of AKI but are of limited success in differentiating severe AKI from less severe forms [50]. Most of these biomarkers are closely linked to a single pathologic process, so that they perform poorly in AKI populations with other pathophysiological mechanisms or heterogeneous origins [49].

Metzger et al. [51] analyzed urine samples from 30 intensive care unit (ICU) patients, 16 of whom developed AKI. CE-MS identified a diagnostic pattern of twenty peptides from six proteins significantly associated with AKI. Peptides of β2-microglobulin, α1 antitrypsin, and albumin were overexpressed, whereas fragments of collagens 1α(I) and 1α(III) and of fibrinogen alpha were downregulated. The marker panel was validated in an independent blinded set of ICU patients (*n* = 20; AUC = 0.84) and in leukemia patients after hematopoietic stem cell transplantation (*n* = 31; AUC = 0.90) [51]. As compared to more established but still experimental biomarkers of AKI such as serum cystatin C, urinary neutrophil gelatinase associated lipocalin, Kidney Injury Molecule-1 (KIM-1) and interleukin 18, the proteomic marker pattern demonstrated a higher prognostic value, detecting AKI up to 5 days in advance of a rise in serum creatinine [51]. In a subsequent case-control validation study in patients after cardiac surgery, the peptide marker pattern showed an AUC of 0.81 for the prediction of AKI [52].

CE-MS, while effective in identifying a urinary peptide marker profile for early AKI diagnosis [51,52], is a highly specialized research tool that is not suitable for daily clinical practice. The MALDI-MS platform, a higher throughput and less skilled approach, seems more suitable than CE-MS for implementation of fast screening assays, being able to provide results within a few hours. In an effort to develop an early predictive test for AKI based on the analysis of urinary peptide biomarkers by MALDI-MS, Carrick et al. [53] collected urine samples from 95 patients with sepsis. A marker panel of 39 peptides proved to be highly predictive of AKI, though only seven peptides had sequence information available. AKI classifier showed a sensitivity of 86% and a specificity of 76%, with an area under the receiver operating characteristics curve of 0.82 [53]. In keeping with a previous study by the same authors [51], increased urinary levels of peptide fragments from alpha-1 antitrypsin and beta-2 microglobulin, and decreased urinary levels of fibrinogen alpha chain, represented early signs of AKI. Excretion of collagen alpha chain-derived peptide fragments was differential, which is indicative of alterations in extracellular matrix turnover, a possible trigger factor in the development of CKD [54]. The MALDI-MS platform can be transferred as a point-of-care test in daily clinical practice for timely detection of septic AKI. A prospective clinical study has been planned to demonstrate any significant benefit from such a biomarker panel [53].

Other clinical studies have identified different biomarkers. The use of serial urinary peptidomics has been employed in subjects undergoing cardiopulmonary bypass surgery, 22 patients with AKI and 22 patients without AKI [55]. This study showed 2 novel peaks (2.43 and 2.78 kDa) to be dominant on postoperative non-AKI urine samples; the 2.78 kDa protein was identified as an active 25-amino acid form of hepcidin, which suggests a novel role for iron sequestration in modulating AKI [55].

There are two clinical trials directly associated with the use of protein biomarkers in acute kidney dysfunction prognosis [56]. In 52 liver cirrhosis patients (trial “Prognostic biomarkers for acute kidney injury in liver cirrhosis”; NCT03156426), measurement of urine and serum levels of KIM-1, as well as urinary liver-type fatty acid-binding protein (L-FABP) and the protein/creatinine ratio, was used to evaluate their prognostic value for AKI occurrence or worsening. Though the trial was completed in November 2017, no results have yet been posted. In the Navigate AKI trial (NCT 02114138; expected completion December 2019), urinary insulin-like growth factor-binding protein 7 (IGFBP7) and tissue inhibitor of metalloproteinases 2 were evaluated as predictors of acute kidney damage after major surgery.

Hopefully, new bioinformatics approaches for the elucidation of the molecular events leading to the development of these molecular species and the mapping of molecular interactions will provide causative insights into the underlying molecular mechanisms and pathways involved in AKI [49].

### 3.3. Kidney Transplantation

Kidney transplantation represents the optimal choice of therapy for end-stage renal disease patients. However, long-term graft function is still limited, mainly due to active or latent rejection, viral infections, and drug toxicity. Identification of post-transplantation complications currently requires an allograft biopsy, which however has monitoring drawbacks and is sometimes ambiguous [57]. There is clearly a need to develop noninvasive accurate diagnostic tests for early diagnosis of graft complications and continuous monitoring over the entire post-transplantation course.

Many proteomic studies have focused on graft rejection, given that, despite improvements in immunosuppressive therapy and patient surveillance after renal transplantation, allograft rejection remains a significant adverse factor for long-term graft survival [58]. Both T cell-mediated rejection and antibody-mediated rejection are leading causes of graft failure.

Using LC-MS/MS analysis, urine samples from kidney transplant patients with biopsy-proven acute rejection showed significantly different levels in a number of proteins compared to patients with stable graft on a biopsy protocol [59]. Specific urinary proteins in patients with acute rejection were primarily related to major histocompatibility complex antigens, the complement cascade, and extracellular matrix. Use of ELISA in an independent set of urine samples cross-validated a subset of proteins (uromodulin, SERPINF1, CD44), showing reduced levels of uromodulin and CD44 and increased levels of SERPINF1 in acute rejection patients [59]. Other authors, using non-targeted MALDI-TOF analysis, detected 630 urinary peptide “features”, among which a panel of 40 down-regulated peptides (all from uromodulin and various collagens) discriminated acute rejection. By integrating urine peptidomic findings (reduced collagen peptides) during graft rejection with those of biopsy gene transcription (increased collagenases and collagen expression, promoting decreased collagen breakdown and increased collagen deposition, respectively), a mechanism of fibrosis caused by acute rejection was proposed [60]. In serial urine samples from 6 patients with biopsy-proven acute rejection during the first post-transplant months, Loftheim et al. [61] used shotgun proteomics to find increased levels of growth factor proteins (vasorin, insulin-like growth factor-binding protein 7, epidermal growth factor, and galectin-3-binding protein), as compared to six age-matched transplant recipients without clinical signs of rejection. Notably, upregulation of proteins was already detectable several days before the acute rejection was clinically suspected by a rise in plasma creatinine [61]. Metzger et al. [62] used CE-MS to investigate the urinary peptidome in subclinical T cell-mediated rejection observed in protocol graft biopsies (in order to detect rejection at an early stage) in comparison to transplant patients with no rejection. The authors identified and included 14 urinary markers in a diagnostic classification model, validated in a blinded independent set (AUC 0.91). Sequence analysis indicated these biomarkers to be collagen fragments (three collagen α-1 fragments and one collagen α-3 fragment), most likely resulting from metalloproteinase 8 proteolysis [62]. This peptide marker set was used in a following prospective, multicenter, blinded phase III trial (NCT01315067) started in 2011 in 12 German transplant centers, enrolling 600 patients with biopsies within the first year of kidney transplantation [63]. The aim of the study was to evaluate whether the marker panel is efficacious in post-transplant patient surveillance for acute rejection in place or as decision guidance for a graft biopsy. The study has been completed but no results have been posted as yet [64].

However, detection of T-cell mediated rejection is not the only need in kidney allograft surveillance [65]. The kidney transplant may be injured by several different causes that require differing approaches for patient management and are indistinguishable by any other available non-invasive test. In an effort to solve this, Sigdel et al. [66] collected renal allograft biopsy-matched urine samples from 396 kidney transplanted patients. This approach led to urinary peptides being identified and validated as biomarkers of specific categories of renal transplant injury, namely acute rejection (11 peptides; AUC 93%), chronic allograft nephropathy (12 peptides; AUC 99%), and BK virus nephritis (12 peptides; AUC 83%). A similar peptidomics urine marker panel could identify different causes of graft injury in patients needing more attention and further workup, without the need for biopsy [66].

When evaluating the urinary peptidomic results, the biological significance of identified molecules and identification of the modulated processes which are involved form a key aspect. In a recent review on proteomic markers for T-cell mediated rejection, use of the pathway- and enzyme reaction-related Reactome information resource revealed processes related to platelet degranulation, lipid digestion, keratan sulphate degradation, antigen presentation and interferon gamma signaling to be directly associated with the input proteins [58]. More recently, Marx et al. [67] used a semantic clustering approach to evaluate the molecular pathway and biological processes specific for different forms of renal allograft disease, including T cell-mediated rejection, antibody-mediated rejection, intestinal fibrosis and tubular atrophy, and polyomavirus-associated nephropathy. By connecting histological and transcriptomic kidney allograft disease characteristics with proteomic biomarker qualification, this approach identified phenotype-specific pathways and associated key molecules that can be used as markers for a given phenotype [67].

### 3.4. Glomerulonephritis

Glomerulonephritis represents one of the most common causes of CKD and requires careful assessment of the disease for prognostic and therapeutic purposes. While many diagnostic tests are currently being used to refine the clinical diagnosis, kidney biopsy remains the gold standard for revealing diagnostic and prognostic histological features. However, it is an invasive procedure, cannot be repeated frequently, and does not provide information on a cellular or signaling pathway level. Urinary proteomic analysis has been proposed as a “liquid biopsy”, which can be used to discriminate various types of kidney diseases [68]. In addition, in contrast to biopsy, peptidome analysis may allow one to evaluate the effects of drugs at the protein signaling pathway level [5]. Though other investigators have diverging opinions as to the value of urinary proteomics versus kidney biopsy in clinics [69], there is agreement that biomarker-based analyses, rather than seeking to discriminate between kidney disease patients and healthy subjects, should focus on correctly diagnosing a specific renal disease among patients with similar manifestations [68,69].

Proteomic approaches have revealed differences in protein expression among many different glomerular diseases [70]. In addition, Rocchetti et al. [71] showed that low urinary levels of kininogen could discriminate between patients with IgA nephropathy, responders or non-responders to therapy with angiotensin-converting enzyme inhibitors. However, those studies suffered from heterogeneity and unduly small patient populations.

The value of urine peptide-based signature panels in the differential diagnosis of certain kidney diseases has been suggested by a recent study of 1180 urine samples [72]. The study cohort included patients with IgA nephropathy (*n* = 179), membranous nephropathy (*n* = 77), focal segmental glomerulosclerosis (*n* = 110), minimal change disease (*n* = 35), lupus nephritis (*n* = 92), vasculitis (*n* = 111), diabetic nephropathy (*n* = 422) and hypertensive nephrosclerosis (*n* = 154). Except for the latter two disorders, all the diagnoses were biopsy-proven. Notably, the study was designed to compare one group with all other kidney disease types, without involvement of healthy individuals. Analysis by CE-MS defined several potential urinary biomarker peptides (ranging from 116 peptides for IgA nephropathy to 619 for diabetes and hypertensive nephrosclerosis), which were combined into support vector machine-based classifiers specific for each kidney disease. The classifiers were validated and proved to discriminate among disease types with good to excellent accuracy. Importantly, several of the biomarkers identified were associated with apparent mechanisms of disease [72] and are thus of potential pathophysiological significance. These findings are promising and beside their diagnostic value, may serve to identify the best-suited therapeutic targets [73] and guide treatment of specific patients. However, they need to be replicated and expanded in additional studies in independent cohorts. One such study is the ongoing PersTIgAN (Personal Treatment in IgA nephropathy) trial, a multidisciplinary transnational research project cofunded by ERA PerMed network [74]. IgA nephropathy, the most common worldwide primary glomerulonephritis, lacks guidance when it comes to predicting therapy response. The PersTIgAN project aims to identify urinary peptides significantly associated with response to specific treatment, using samples from patients with known outcome, as well as to develop and test an algorithm to predict response based on those urinary peptides. If the algorithm shows significant benefit, then a clinical trial is planned randomizing incident patients predicted responsive to therapy into two groups, one treated with immunosuppressive and supportive therapy and the other with supportive treatment only [74]. This project represents a further effort to personalize intervention and support implementation in routine care.

## 4. Conclusions

For better management of kidney diseases, we urgently need to develop highly accurate biomarkers for use in clinics. Proteins and peptides are the main functional and structural units of the cell and hence of great biomarker potential, since qualitative and quantitative differences in the proteome and peptidome composition reflect pathologic conditions. Based on current evidence, mostly in the context of CKD, urinary peptidomics may bring a significant improvement in kidney disease management by supporting earlier and more accurate detection, prognostic assessment, and prediction of response to treatment. Peptidomics/proteomics is expected to develop into the key technology to guide personalized intervention, and CKD273 classifiers may serve as a first example for personalized medicine in nephrology [8].

However, despite the abundance of publications, the clinical translatability of peptidomic studies, already appearing in the literature, has some major limitations which should be considered. One major argument against implementation of urinary peptidomics in daily clinical practice is the lack of guidance on how to place the biomarkers in the clinical context [5]. Clinical results have so far usually been obtained in studies differing in design and based on small cohorts; hence the need for validation by adequate investigations. Several European Union-funded studies are ongoing (BIOMARGIN, iMODE-CKD, EURonOmics), aiming to provide evidence for the clinical utility of urinary peptidome analysis in kidney disease. Much current effort, including the development of improved omics-study designs, and multicenter validations with multidisciplinary teams possessing complementary knowledge, holds out promise of a more widespread and effective clinical use of peptidomics [28].

Sharing of data obtained in peptidomic studies among the scientific community is also of critical importance, since it will ensure data are available, encourage collaboration, and facilitate integration [75]. Specialized scientific databases greatly help to facilitate this process [76]. In the field of nephrology, omics databases include “the Kidney and Urinary Pathway Knowledge Base” [77], the “Human Urinary Proteome Fingerprint Database” [78], and the “Chronic Kidney Disease database” [79]. More recently, a publicly available database platform that solely collects peptidomic and proteomic datasets manually extracted from published studies relative to CKD has been set up [80]. The peptiCKDdb resource (www.peptiCKDdb-com) is expected to facilitate modeling of molecular mechanisms underlying CKD and identification of relevant biomarkers [80].

Additional issues blocking clinical implementation regard acceptance by the medical community and the cost-effectiveness of the approach. Urinary proteome analysis is complex, requires expertise, and is not without significant costs. The current technological platforms have been showing limitations in their implementation in laboratory medicine facility, providing ground for the development of centralized facility service over the wide territorial area. If this model proves to be cost effective in terms of laboratory production, it also demonstrates that the current results are not of direct use in taking an immediate medical decision as often required in kidney disease healthcare. The development of more robust analytical platforms is a fundamental prerequisite to explore effectively the clinical application of peptidome analysis, which necessarily requires the molecular discriminatory capacity of mass spectrometer analysers.

However, a recent health economy assessment indicates that early detection in type 2 diabetic patients, as achievable by peptidomic analysis, is cost-effective [81]. In a Markov model it was in fact demonstrated that annual use of the CKD273 classifier for early assessment and modeled interventions, though more expensive than albuminuria, yields more quality-adjusted life years [81].

In conclusion, urinary proteomics is one such promising technology that could help to personalize treatment in patients suffering from kidney disease [6], though its translation into day-to-day practice is lagging behind. Recent technical advances as well as certain current and projected ventures, may ultimately clinch the use of urinary peptidomics in the near future as a major part of kidney disease management.
